# Beta-blockade is not associated with improved outcomes in isolated severe extracranial injury: an observational cohort study

**DOI:** 10.1186/s13049-021-00947-6

**Published:** 2021-09-08

**Authors:** Lin Sadi, Gabriel Sjölin, Rebecka Ahl Hulme

**Affiliations:** 1 Department of Surgery, Capio St Görans Hospital, Stockholm, Sweden; 2 Department of Surgery, Örebro University Hospital, Örebro, Sweden; 3 School of Medical Sciences, Örebro University, Örebro, Sweden; 4 Division of Trauma and Emergency Surgery, Department of Surgery, Karolinska University Hospital, Stockholm, Sweden

**Keywords:** Beta blockers, Traumatic injury, Mortality

## Abstract

**Background:**

There is evidence supporting the use of beta-blockade in patients with traumatic brain injury. The reduction in sympathetic drive is thought to underlie the relationship between beta-blockade and increased survival. There is little evidence for similar effects in extracranial injuries. This study aimed to assess the association between beta-blockade and survival in patients suffering isolated severe extracranial injuries.

**Methods:**

Patients treated at an academic urban trauma centre during a 5-year period were retrospectively identified. Adults suffering isolated severe extracranial injury [Injury Severity Score (ISS) ≥ 16 with Abbreviated Injury Score of ≤ 2 for any intracranial injury] were included. Patient characteristics and outcomes were collected from the trauma registry and hospital medical records. Patients were subdivided into beta-blocker exposed and unexposed groups. Patients were matched using propensity score matching. Differences were assessed using McNemar’s or paired Student’s t test. The primary outcome of interest was 90-day mortality and secondary outcome was in-hospital complications.

**Results:**

698 patients were included of whom 10.5% were on a beta-blocker. Most patients suffered blunt force trauma (88.5%) with a mean [standard deviation] ISS of 24.6 [10.6]. Unadjusted mortality was higher in patients receiving beta-blockers (34.2% vs. 9.1%, *p* < 0.001) as were cardiac complications (8.2% vs. 1.4%, *p* = 0.002). Patients on beta-blockers were significantly older (69.5 [14.1] vs. 43.2 [18.0] years) and of higher comorbidity. After matching, no statistically significant differences were seen in 90-day mortality (34.2% vs. 30.1%, *p* = 0.690) or in-hospital complications.

**Conclusions:**

Beta-blocker therapy does not appear to be associated with improved survival in patients with isolated severe extracranial injuries.

## Introduction

Several studies have observed a survival benefit in patients suffering traumatic brain injury (TBI) and who are on concomitant beta-blocker therapy [1–7]. The protective use of beta-blockade in the context of major physiological stress is further supported by observed reductions in morbidity and mortality following emergency cardiac and non-cardiac surgery in patients receiving perioperative beta-blocker treatment [8, 9]. These findings have been linked to the inhibition of the stress responses initiated by significant tissue injury such as major surgery and traumatic injury.

Physiological stress is characterised by an increase in the secretion of catecholamines and activation of the sympathetic nervous system. Circulating catecholamines are a vital component for regulating multiple organ and inflammatory systems [10]. The excessive activation of adrenergic receptors has been associated with an increased risk of death [11, 12] mediated by cardiac, pulmonary, and immunological dysfunction [13–15]. It is believed that the catecholamine surge may worsen secondary tissue injury by causing vasoconstriction and subsequent ischemia [16, 17] and result in the development of extracranial organ dysfunction [18–20].

While there is research supporting the role of beta-blocker therapy in the context of traumatic brain injury, only one retrospective study has shown that beta-blocker therapy can reduce mortality in critically injured patients without TBI [21]. More evidence in the context of beta-blocker use in extracranial trauma is therefore required. This retrospective cohort study was carried out to investigate whether beta-blocker therapy can improve clinical outcomes in trauma patients suffering severe extracranial injuries. The study hypothesis was that there would be improved survival in patients on a regular beta-blocking agent.

## Methods

### Patient selection

This is a retrospective single centre observational cohort study. The Declaration of Helsinki was adhered to. Ethical approval was granted from the regional ethics review board and the local hospital trauma committee (reference number 2015/961-31/4). The trauma registry of Karolinska University Hospital, an academic urban trauma centre in Stockholm, Sweden, was queried for patients admitted between 1/2007 and 12/2011. The inclusion of patient information in the registry and the possibility of registry-based research are dependent on patient consent on an opt-out basis. The need for further patient consent was waived by the regional ethics review board. All adult patients (age 18 years and older) were enrolled. Patients were screened for type and severity of their injuries and only those with isolated severe extracranial injuries met inclusion criteria. Isolated severe extracranial injuries were defined as an overall Injury Severity Score (ISS) of at least 16 with an Abbreviated Injury Scale (AIS) score of two or less for any intracranial injury suffered.

Patient characteristics and clinical outcomes were obtained from the hospital’s trauma registry and from in-hospital patient electronic medical records. Collected variables included age, sex, injury mechanism, admission Glasgow Coma Scale (GCS) score, admission systolic blood pressure (SBP), ISS, surgery, beta-blocker administration, need for dialysis, intensive care unit (ICU) admission and total hospital length of stay (LOS). The Charlson Comorbidity Index (CCI) was obtained from the electronic medical records and was used to measure the burden of disease prior to the experienced trauma. Recorded in-hospital morbidity included infections, respiratory complications (e.g. re-intubation, tracheostomy) and cardiac complications (e.g. arrhythmias, acute cardiac failure, myocardial infarction). In-hospital mortality and mortality up to 90 days were recorded. Patients declared dead on arrival were excluded.

### Beta-blocker therapy

Information about beta-blocker therapy was sought from the drugs registry of the National Board of Health and Welfare. Unique national personal identity numbers were used to match patients to the national database for drug prescriptions. All medical prescriptions in Sweden are registered in this database including type, dosage and date of issuing and collection. Indication for use is not collected in the drugs registry. Information about beta-blockers were collected within one year of the date of hospital admission for each patient in order to capture patients on beta-blockers with long-term prescriptions issued for several months at a time. Only patients with an issued beta-blocker prescription that had also been collected prior to the trauma met the criteria for beta-blocker exposed cases. Hospital medical records were also reviewed to ensure that patients on preinjury beta-blockers were not discontinued on their beta-blocker during admission. Patients were subsequently divided into beta-blocker exposed and unexposed groups.

### Statistical analysis

The primary outcome of interest was mortality within 90 days of the traumatic injury and the secondary outcome of interest was in-hospital morbidity. For the purpose of statistical analysis, certain variables were dichotomised using clinically relevant cut-off points (SBP < 90 mmHg vs. ≥ 90 mmHg and ISS 16–24 vs. 25–49 vs. 50–74). Patient demographics and clinical outcomes were compared between beta-blocker exposed and unexposed groups. The Chi-square test with continuity correction, or Fisher´s exact test when appropriate, were used to analyse categorical variables. The Student´s *t* test, or Mann Whitney U test when appropriate, were used for continuous variables. Results are reported as mean ± standard deviation (SD) or median with interquartile range [IQR] for continuous variables and as percentages for categorical variables.

Due to a larger number of confounders within the cohort, beta-blocker exposed patients were matched in a 1:1 ratio to beta-blocker unexposed patients through propensity score matching. Included in the propensity score model were the following variables: age, injury mechanism, ISS, SBP, CCI and surgery required or not. Propensity scores were calculated using binary logistic regression. Each beta-blocker exposed patient was matched to a beta-blocker unexposed patient within a 0.0135 caliper of propensity without replacement and the caliper was equal to one quarter of a standard deviation of the logit of the propensity score. Group differences were then assessed using McNemar’s test for categorical variables and the paired Student’s *t* test or Wilcoxon signed rank test for continuous variables. Results were considered statistically significant when the p-value was below 0.05. Data were analysed using the Statistical Package for Social Science (SPSS Windows) version 21.0 (SPSS Inc., Chicago, IL).

## Results

A total of 698 patients met the study inclusion criteria over the five-year study period. The mean age of the total population was 46.0 (19.4) years and there was a predominance of the male sex with 79.4% (n = 554). The most common injury mechanism was blunt force trauma (88.5%, n = 618), median ISS was 21 [17–27] and 73.9% (n = 516) required surgery for their traumatic injury (Table 1). The overall mortality for the total cohort was 11.7% (n = 82). Overall, 10.5% (n = 73) of the study population was exposed to regular beta-blocker therapy prior to admission. Metoprolol was the most common type of beta-blocker prescribed in 69.9% (n = 51) of cases (Table 2).

**Table 1:** Demographics of the total cohort prior to subgrouping based on beta-blocker exposure

Variable	Total cohort (n = 698)
Beta-blocker therapy, % (n)	10.5 (73)
Age, mean [SD] years	46.0 [19.4]
Male sex, % (n)	79.4 (554)
GCS > 8, % (n)	86.2 (602)
SBP < 90 mmHg, % (n)	12.3 (86)
Penetrating injury, % (n)	11.5 (80)
ISS, mean [SD]	24.6 [10.6]
ISS, median [IQR]	21 [17–27]
ISS 16–24, % (n)	63.9 (446)
ISS 25–49, % (n)	32.2 (225)
ISS 50–74% (n)	2.0 (14)
ISS 75, % (n)	1.9 (13)
CCI, mean [SD]	1.1 (1.7)
CCI, median [IQR]	0 (0–2)
Surgery required, % (n)	73.9 (516)

SD, standard deviation; IQR, interquartile range; GCS, Glasgow Coma Scale; SBP, Systolic Blood Pressure; ISS, Injury Severity Score; CCI, Charlson Comorbidity Index

**Table 2:** Beta-blocker type

Drug name	Prevalence % (N)	Receptor selectivity
Sotalol	2.7% (2)	Non-selective
Bisoprolol	8.2% (6)	Selective
Propranolol	6.9% (5)	Non-selective
Atenolol	9.6% (7)	Selective
Carvedilol	2.7% (2)	Alpha and beta-receptor selectivity
Metoprolol	69.9% (51)	Selective

Prior to propensity score matching there were some significant differences between patient groups. Patients receiving beta-blocker therapy were significantly older (69.5 [14.1] vs. 43.2 [18.0] years, *p* < 0.001), required emergency surgery to a lesser degree (61.6% vs. 75.6%, *p* = 0.015) and contained fewer men (67.1% vs. 80.8%, *p* = 0.010) than patients unexposed to beta-blockers. The percentage of patients with a CCI of zero was 64.3% in beta-blocker unexposed compared to only 8.2% in beta-blocker exposed (*p* < 0.001) (Table 3). No significant differences were seen with regards to admission GCS, hypotension on admission, ISS score and injury mechanism (Table 3). SBP on admission was also analysed as a continuous variable to see whether pre-admission beta-blocker use resulted in significantly lower systolic pressures on admission. No statistically significant difference was found, with beta-blocked patients demonstrating a median [IQR] SBP of 140 [116–165] compared to beta-blocker unexposed patients with a corresponding value of 131 [110–150] (*p* = 0.061).

**Table 3:** Demographics of the subgroups based on beta-blocker exposure prior to and after propensity score matching

Variable	Before matching			After matching		
	BB^+^ (n = 73)	BB^–^ (n = 625)	*p*	BB^+^ (n = 73)	BB^–^ (n = 73)	*p*
Age, mean [SD] years	69.5 [14.1]	43.2 [18.0]	< 0.001	69.5 [12.3]	70.2 [14.6]	0.628
Male sex, % (n)	67.1 (49)	80.8 (505)	0.010	67.1 (49)	68.5 (50)	1.000
GCS > 8, % (n)	91.8 (67)	85.6 (535)	0.204	91.8 (67)	84.9 (62)	0.302
SBP < 90 mmHg, % (n)	12.3 (9)	12.3 (77)	1.000	12.3 (9)	13.7 (10)	1.000
Penetrating injury, % (n)	6.8 (5)	12.0 (75)	0.266	6.8 (5)	5.5 (4)	1.000
ISS, mean [SD]	23.7 [7.8]	24.7 [10.9]	0.482	23.7 [12.3]	23.5 [9.8]	0.894
ISS 16–24, % (n)	64.4 (47)	63.8 (399)	1.000	64.4 (47)	71.2 (52)	0.442
ISS 25–49, % (n)	32.9 (24)	32.7 (201)	1.000	32.9 (24)	23.3 (17)	0.210
ISS 50–74, % (n)	2.7 (2)	1.9 (12)	0.650	2.7 (2)	4.1 (3)	1.000
CCI 0, % (n)	8.2 (6)	64.3 (401)	< 0.001	8.2 (6)	4.1 (3)	0.375
CCI 1, % (n)	13.7 (10)	14.9 (93)	0.924	13.7 (10)	15.1 (11)	1.000
CCI 2, % (n)	17.8 (13)	8.0 (50)	0.011	17.8 (13)	19.2 (14)	1.000
CCI 3, % (n)	17.8 (13)	4.8 (30)	< 0.001	17.8 (13)	15.1 (11)	0.791
CCI 4, % (n)	15.1 (11)	5.0 (31)	0.002	15.1 (11)	20.5 (15)	0.503
CCI 5, % (n)	12.3 (9)	1.8 (11)	< 0.001	12.3 (9)	15.1 (11)	0.815
CCI 6, % (n)	4.1 (3)	0.6 (4)	0.028	4.1 (3)	5.5 (4)	1.000
CCI 7, % (n)	2.7 (2)	0.5 (3)	0.088	2.7 (2)	2.7 (2)	1.000
CCI 8, % (n)	6.8 (5)	0.2 (1)	< 0.001	6.8 (5)	1.4 (1)	0.125
CCI 9, % (n)	1.4 (1)	0 (0)	0.105	6.8 (5)	1.4 (1)	0.125
Surgery required, % (n)	61.6 (45)	75.6 (471)	0.015	53.4 (39)	54.8 (40)	1.000

Patients were matched using propensity score matching. Variables matched included age, injury mechanism, ISS, SBP, CCI and surgery required or not

SD, standard deviation; GCS, Glasgow Coma Scale; SBP, Systolic Blood Pressure; ISS, Injury Severity Score; CCI, Charlson Comorbidity Index

Beta-blocker positive patients suffered more cardiac complications (8.2% vs. 1.4%, *p* = 0.002) but no significant differences were observed in respiratory events, infections, the need for dialysis or in ICU and hospital LOS (Table 4). Unadjusted mortality was higher in patients who received beta-blocker treatment (34.2% vs. 9.1% %, *p* < 0.001).

**Table 4:** Clinical outcomes of the subgroups based on beta-blocker exposure prior to and after propensity score matching

Variable	Before matching			After matching		
	BB^+^ (n = 73)	BB^–^ (n = 625)	*p*	BB^+^ (n = 73)	BB^–^ (n = 73)	*p*
Infection, % (n)	30.1 (22)	27.7 (173)	0.767	30.1 (22)	27.4 (20)	0.851
Cardiac complication, % (n)	8.2 (6)	1.4 (9)	0.002	8.2 (6)	4.1 (3)	0.453
Respiratory complication, % (n)	6.8 (5)	5.3 (33)	0.583	6.8 (5)	8.2 (6)	1.000
Dialysis, % (n)	4.1 (3)	1.8 (11)	0.173	4.1 (3)	1.4 (1)	0.625
ICU LOS, mean [SD]	2.8 [6.1]	2.8 [5.4]	0.980	2.8 [8.7]	2.8 [8.7]	0.852
Hospital LOS, mean [SD]	14.6 [18.8]	15.2 [16.8]	0.771	14.6 [23.1]	13 [15]	0.569
*Care level at day 30*						
1, % (n)	16.4 (12)	16.2 (101)	1.000	13.7 (10)	15.1 (11)	1.000
2, % (n)	5.5 (4)	12.6 (79)	0.110	17.8 (13)	15.1 (11)	0.791
3, % (n)	1.4 (1)	0.5 (3)	0.358	15.1 (11)	20.5 (15)	0.503
4, % (n)	46.6 (34)	61.0 (381)	0.025	12.3 (9)	15.1 (11)	0.815
In-hospital mortality, % (n)	28.8 (21)	8.2 (51)	< 0.001	28.8 (21)	24.7 (18)	0.690
30-day mortality*, % (n)	30.1 (22)	8.5 (53)	< 0.001	30.1 (22)	26 (19)	0.701
90-day mortality**, % (n)	34.2 (25)	9.0 (56)	< 0.001	34.2 (25)	30.1 (22)	0.690

Patients were matched using propensity score matching. Variables matched included age, injury mechanism, ISS, SBP, CCI and surgery required or not

SD, standard deviation; GCS, Glasgow Coma Scale; SBP, Systolic Blood Pressure; ISS, Injury Severity Score; CCI, Charlson Comorbidity Index. Care level at day 30 is defined accordingly: 1, in hospital; 2, rehabilitation center; 3, care home; 4, at home

*30-day mortality includes all deaths up to 30 days after suffered trauma

**90-day mortality includes all deaths up to 90 days after suffered trauma (includes all patients that count towards 30-day mortality)

Following propensity score matching, there were no statistically significant differences in patient characteristics between subgroups (Table 3). In addition, the previously observed differences in cardiac complications and mortality were eliminated. After matching, no statistically significant differences could be seen between the beta-blocker exposed and unexposed groups in either in-hospital deaths (28.8% vs. 24.7%, *p* = 0.690) or long-term mortality at 90-days post-trauma (34.2% vs. 30.1%, *p* = 0.690) (Table 4).

## Discussion

Many studies have been published on the subject of beta-blockade and traumatic brain injury, where the majority of scientific trauma literature support a protective role between the drug and survival [1–5]. Systemic catecholamines rise at the onset of traumatic tissue injury which generates a hyperadrenergic state. The catecholamine surge, measured at time of hospital admission, has a strong relationship with the extent of tissue injury [12]. In the context of TBI, the catecholamine surge leads to vasoconstriction of cerebral vessels which decreases perfusion and oxygenation. Consequently, in some centres, beta-blockers are used clinically in a recognized method of intracranial pressure-targeted management, the so called “Lund concept”, in order to reduce cerebral strain and optimize brain recovery. The aim of the Lund concept is to restore normal intracranial physiology after TBI [22]. It has shown improved outcomes compared to other intracranial pressure management regimes [23]. In vivo, propranolol administration in animal models demonstrate increased cerebral perfusion and decreased hypoxia [24]. The potential therapeutic role of beta-blockade in TBI has therefore led to the hypothesis of a similar link between beta-blockers and extracranial traumatic injuries. The scientific evidence for this, however, is very limited.

Extracranial effects of the hyperadrenergic state following traumatic injury are complex but include cardiac stress, hypermetabolism, immunosuppression and coagulopathy. Catecholamines play a direct role in cardiac strain and lead to a rise in heart rate and contractility which increase the oxygen demands of the myocardium. In turn, this increases the risk of myocardial ischaemia and tachyarrhythmias. The risk of suffering an acute myocardial infarction is not only increased in patients who suffer direct trauma to the heart but has also been demonstrated in the context of abdominal and pelvic trauma [25]. In addition to its strain on the cardiovascular system, catecholamine release encourages hypermetabolism and catabolism through the mobilization of substrates from fat, proteins and carbohydrate stores [26]. Thus, the hypercatabolic state in trauma patients may result in muscle atrophy, weight loss and ketogenesis with resulting exacerbation of acidaemia [27]. This increases the risk of in-hospital complications and could impede recovery.

Furthermore, traumatic tissue injury results in the release of neutrophils, macrophages and cytokines which leads to proinflammatory consequences [10]. Exacerbation of inflammatory responses to traumatic injury may lead to systemic inflammatory response syndrome (SIRS) and subsequent multiorgan failure. Attempts to control inflammatory hyperactivity through immunosuppression leads to an increased risk of infections [28]. Finally, hyperactivation of the sympatho-adrenal axis has been linked to vascular dysfunction where circulating catecholamines demonstrate an independent association with endotheliopathy and mortality [29]. Blockade of adrenoceptors has shown to attenuate many of these detrimental processes and has therefore been put forward as a potential method of improving patient outcomes following both traumatic and surgical stress.

To the best knowledge of the authors, only one study exists that corroborates this in the context of trauma care following severe extracranial injury. In 2012, Bukur and colleagues published a retrospective study that demonstrated a 63% risk reduction in mortality in critically injured patients without head injuries who received beta-blockade within 30 days of admission to intensive care. Despite demonstrating a lower mortality rate for patients on beta-blockers (11.2% vs. 19.3%), rates of in-hospital complications such as acute respiratory distress syndrome, acute renal failure and multiple organ failure were all higher in beta-blocked patients [21]. The difference in effect on mortality and complications does bring about a certain level of uncertainty. Selection bias may explain this difference where in-hospital beta-blocker administration is simply a substitute for a more circulatory stable trauma patient. In addition, patients were not matched, and regression analysis only was used in order to compensate for the likely wide injury panorama, and thereby heterogeneity, seen in this patient group.

In contrast, after matching for clinically relevant variables, no statistically significant relationship was found between preinjury beta-blockade and 90-day survival in patients suffering isolated severe extracranial injury in the current study. Unadjusted mortality was higher in patients who received beta-blocker treatment (34.2% vs. 9.1% %, *p* < 0.001). This is likely to reflect the higher comorbidity burden and increased mean age of this group. Following propensity score matching, there was no difference between ICU LOS, total hospital LOS and in-hospital complications. This suggests that any potential beneficial effects of beta-blockers are unable to surmount the impact of biological factors such as age and the level of comorbidity burden.

Similar results to those of the current study were demonstrated by Hendrick et al. who found no benefit to beta-blockade in multi-trauma patients without significant head injury after adjusting for potential confounding patient variables [30]. The absence of a relationship between preinjury beta-blocker therapy and survival in patients with traumatic injury were also demonstrated by Eriksson et al. [31]. In addition, Neideen et al. found that preinjury beta-blockade increased the risk of death in the elderly trauma population [32]. These findings were corroborated by Evans et al. who demonstrated increased mortality in geriatric trauma patients on cardiac medications such as beta-blockers [33]. This finding suggests that the study of beta-blocker therapy in isolation may be somewhat incomplete. Patients on beta-blockers are often also prescribed other cardiac drugs such as anti-hypertensive treatments, which could affect outcomes. The inclusion of beta-blockers only must, therefore, be recognised as a limitation of the current study and future studies should seek to expand the classes of drugs included. Such an expansion could address whether the suppression of sympathetically induced hypertension is an important mechanism, distinct from the direct effect of beta-blockade on the sympathetic drive. Interestingly, Ferraris and colleagues showed that trauma patients on preinjury beta-blockade had an equivalent increase in the risk of suffering a fatal outcome to patients with pre-existing congestive cardiac failure or those on preinjury warfarin [34]. These studies did, however, not distinguish between intracranial and extracranial injuries which may make the interpretation and comparison of results more difficult.

In the current study, cardiac complications occurred in 8.2% of beta-blocked patients compared to 1.4% of those unexposed to the drug. This is expected since patients on beta-blockers are more likely to have a higher cardiac risk index compared to patients who do not have conditions that require a beta-blocking agent. This difference was eliminated when adjusting for potential confounders. This is an interesting observation since beta-blocker therapy is often considered a sign of a patient with an increased cardiac risk. Hence, the lack of an association between cardiac complications and beta-blocker therapy could indicate that the continuation of preinjury beta-blockade during hospital admission is important in order to counteract the increased cardiac risk of patients already on beta-blockers.

There are several limitations to the present study, many due to the retrospective nature and registry-based design. Firstly, registers run the risk of incomplete data collection and undiagnosed injuries are a possibility. Secondly, the propensity score matching of the current study was based on ISS in order to reflect the overall injury burden of the patient. While matching based on AIS would have offered a more detailed matching process based on specific organ injuries, it is the view of the authors that matching based on both variables would have risked resulting in over-matching. Thirdly, we were unable to control for factors such as beta-blocker indication, dosage and administration timings as well as sporadic in-hospital dosage, which has previously shown association with better survival [1]. In addition, adjustments for the specific type or dose of beta-blocker were not possible due to the limited cohort size. Different types of beta-blockers may demonstrate different effects on trauma-induced stress, as may increasing doses of the same type of beta-blocker. In addition, the receptor selectivity of the beta-blocker may also be of relevance. Although an overwhelming majority (69.9%) of patients were on metoprolol, a cardioselective beta-blocker, the current study includes five additional types of beta-blockers that vary in selectivity and dosage. It is, therefore, possible that some beta-blockers may be more or less favourable in the context of reducing trauma-induced stress reactions, which may affect the demonstrated results, but this study is unable to draw any conclusions on this matter. Future studies should consider this limitation and the authors suggest that patient inclusion is limited to those on the same type of beta-blocker and similar dose.

## Conclusions

The current study could not find any beneficial effects of beta-blocker therapy on outcomes following isolated severe extracranial injury. This suggests that the clinical pathway of multi-trauma patients is complex and that there is no quick fix in improving survival. The lack of scientific literature investigating the relationship between beta-blockade and trauma victims without significant head injuries is evident. The authors hope that this study will encourage more studies on the subject and pave the way for further randomized trials in order to determine the possible therapeutic role of beta-blockade in multi-trauma care.

## Data Availability

The datasets used and analysed during the current study may be available from the corresponding author on reasonable request and with the appropriate ethics approvals.

## Abbreviations

TBI: Traumatic brain injury
ISS: Injury Severity Score
AIS: Abbreviated Injury Scale
GCS: Glasgow Coma Scale
SBP: Systolic blood pressure
ICU: Intensive Care Unit
CCI: Charlson Comorbidity Index
SD: Standard deviation
IQR: Interquartile range
LOS: Length of stay
SIRS: Systemic Inflammatory Response Syndrome
